# Analysis of nutrition status and family compliance in children with cerebral palsy

**DOI:** 10.3389/fped.2026.1788864

**Published:** 2026-07-13

**Authors:** Xuemei He, Yangping Zhang, Jing Wang, Haoyu Huang, Xueyan He, Zhuo Zou, Nan Zheng, Shuyue Yin, Jian Ren, Yiqing Zhou, Xuanlan Cao, Yun Liu

**Affiliations:** 1 Department of Rehabilitation, Kunming Children's Hospital, Kunming Medical Unicersity, Kunming, Yunnan, China; 2 Women's Psychiatric Department, Second People's Hospital of Pu ‘er, Pu ‘er, Yunnan, China

**Keywords:** cerebral palsy, dysphagia, family compliance, malnutrition, nutritional assessment

## Abstract

**Background:**

The overall nutritional status of children with cerebral palsy in China is not optimistic. Although some current studies have focused on the nutritional status of children with cerebral palsy or the compliance of their family members, there are relatively few studies that conduct a systematic analysis by combining the two. This study conducts an investigatory analysis of the nutritional status of children with cerebral palsy and the compliance of their family members, aiming to provide a basis for strengthening the nutritional management of children with cerebral palsy.

**Methods:**

This cross-sectional study investigated the nutritional status of 58 children with cerebral palsy treated at the Rehabilitation Department of Kunming Children's Hospital (January 2023–December 2024), alongside their families' compliance with nutritional management. Data collection included anthropometric measurements (weight, height, head circumference, triceps and subscapular skinfold thickness, mid–upper arm circumference), biochemical indicators (serum albumin, total protein, and 25-hydroxyvitamin D), family questionnaire survey and the SGNA. The rank - sum test for two - sample comparison was employed to compare the serum total protein and albumin levels between the control group and the diseased group. The weighted kappa was utilized to analyze the consistency of the nutritional grades between SGNA and ASPEN *Z*-score.

**Result:**

1. Malnutrition prevalence was strikingly high at 50.00%, with all median growth *Z*-scores falling below normal reference levels. 2. Dysphagia affected nearly half of the children (48.72%). 3. The SGNA demonstrated strong concordance with the WHO malnutrition grading system (*κ* = 0.741; 95% CI: 0.623–0.859; *p* < 0.01). 4. Biochemical deficits included markedly lower serum albumin and total protein compared to controls (*P* < 0.05). 5. Parental perceptions varied: 52.63% believed cerebral palsy is linked to malnutrition, while 47.36% did not; only 35% perceived their child as malnourished vs. 65% who did not. Merely 12.28% of parents reported regular nutritional assessments for their children.

**Conclusion:**

Children with cerebral palsy exhibit a high prevalence of malnutrition, yet family compliance with nutritional management is suboptimal. Future interventions should focus on strengthening parental health education and streamlining nutritional assessment processes to improve management outcomes.

## 1 Introduction

Cerebral palsy (CP) is an early-onset lifelong neurodevelopmental condition characterized by limitations in activity due to impaired development of movement and posture, manifesting as spasticity, dystonia, choreoathetosis, and/or ataxia. It results from maldevelopment attributed to dysplasia of or injury to the fetal or infant brain that is not degenerative, although the manifestations may change with age. The phenotype of CP is complex and heterogeneous, with each person experiencing a unique presentation. In addition to motor dysfunction, people with CP frequently encounter primary and secondary impairments across various areas of development and functioning, which can significantly impact their participation in daily life (1). CP is the most common major disabling motor disorder of childhood with an estimated prevalence of 2–3 cases per 1,000 births (2).

The prevalence rate of cerebral palsy among children aged 0–6 in China is 0.23% (3). Protein-energy malnutrition is one of the common comorbid disorders in children with cerebral palsy. According to different clinical manifestations, it can be classified into three types: underweight, wasting, and stunting. The incidence rate of Protein-energy malnutrition in children with cerebral palsy can be as high as 76.6% (4). Prolonged malnutrition can further lead to developmental delay, anemia, reduced immune function, increased risk of infection, poor brain development, impaired cognitive function, gastrointestinal diseases, and osteoporosis, which significantly impact the quality of life of both the children and their families. Additionally, children with cerebral palsy who suffer from malnutrition are prone to developing pressure sores, pneumonia, and urinary tract infections. Moreover, it also significantly increases the risk of postoperative complications (5). Although nutrition is extremely important for children with cerebral palsy, the nutritional status of these children is still not satisfactory. The compliance of the main caregiver of the family with nutritional follow-up and intervention is an important influencing factor. The compliance of the main caregiver of the family with nutritional follow-up and intervention is an important influencing factor. The nutritional intervention for children with cerebral palsy has the characteristics of long-term implementation at home and requiring the full cooperation of the main caregiver throughout the process. The main caregiver of the family, as the direct executor of the child's diet plan and the main person responsible for nutritional intake, their participation in nutritional follow-up and the compliance of their execution of the intervention plan are the key variables that determine whether the nutritional intervention can be implemented and whether it can achieve the expected effect.

At present, there are relatively few studies that combine the nutritional status of children with cerebral palsy and the compliance of foster parents. The lack of a systematic assessment and management framework has led to the lack of a basis for the implementation of clinical nutritional intervention, resulting in inconsistent nutritional intervention effects. This study is the first to combine the nutritional assessment of children with cerebral palsy with the compliance of the main foster parents, aiming to emphasize the importance of the compliance of the main foster parents in improving the nutritional status of children with cerebral palsy, and providing practical guidance for enhancing the effectiveness of nutritional intervention for children with cerebral palsy and promoting their growth, development and functional recovery.

## 2 Materials and methods

### 2.1 Study population

From January 2023 to December 2024, random sampling was conducted using a random number method, and 58 patients with cerebral palsy who visited the Children's Hospital in Kunming were selected (24, 25). The inclusion criteria are: ① Age 1–10 years; ② Patient and family willingness to cooperate with physical examinations andquestionnaires; ③ Signed informed consent. Exclusion criteria included:

① Severe respiratory, digestive, or circulatory diseases; ② Severe wasting diseases such as advanced liver disease, kidneydisease, tuberculosis, or hematological disorders; ③ Severe surgical complications;

④ Requirementfor long-term parenteral nutrition. The normal control group was randomly sampled using a random number method, and 42 healthy individuals who visited Kunming Children's Hospital for physical examinations were selected. After consulting with statistical experts, this study used the PASS software to calculate the sample size. Serum Total Protein, Albumin, and 25-Hydroxy-Vitamin D Levels were selected as the calculation indicators. With a standard deviation *σ* = 2, an allowable error *δ* = μ1–μ2 = 1, a test level *α* = 0.05, and a test power 1 - *β* = 0.8, the calculated sample size was 86 people. Considering a 10% dropout rate, the minimum sample size required for both groups was 94 cases. The subjects were all fed orally and did not receive nasal feeding or parenteral nutrition, they have no particular dietary habits. This study was approved by the Ethics Committee of Kunming Children's Hospital (Kunerlun Approval No. 2020-03-127-K01).

### 2.2 Physical development indicators

Nutritional assessment included WHO growth and development *Z*-scores (weight-for-age, height-for-age, weight-for-height, and BMI-for-age), triceps and subscapular skinfold thickness, mid-upper arm circumference, head circumference, and calf circumference. Measurements were conducted by trained medical staff, with each value recorded three times and averaged to an accuracy of 0.1 units. For children with severe CP under 2 years old, recumbent length was measured. For those ≥2 years old unable to stand due to joint contractures, limb deformities, or scoliosis, height was estimated using the Stevenson formula (6), that is, height (cm) = (tibial length × 3.26) + 30.8. Method for measuring tibial length: The child is seated with feet flat on the floor and the lower leg perpendicular to the ground. With the knee flexed, the examiner palpates the concavity between the femur and tibia on the medial side of the patellar ligament, locates the upper edge of the medial condyle, then the lower edge of the medial malleolus. The distance between these two points is measured with a flexible tape.

### 2.3 The gross motor function classification system

In this study, the Gross Motor Function Classification System (GMFCS) grading criteria refer to the 2007 revised version designed by McDowell et al. (7). This scheme is a five - level grading system used to describe the gross motor function of children or adolescents with cerebral palsy. The content of the scheme is based on the assessment of the children's ability of independent movement, with a focus on aspects such as sitting, walking, and wheelchair - assisted movement. The specific grading is determined according to the children's ages: less than 2 years old, 2–4 years old, 4–6 years old, 6–12 years old, and 12–18 years old. Within each age group, individuals are classified into five levels (I–V) based on the severity of motor impairment from low to high: Level I, individuals can walk without restrictions but are limited in performing high - difficulty and skill - demanding motor skills; Level II, individuals can walk without the use of assistive devices, but their walking is restricted outdoors and in the community; Level III, individuals need to use assistive devices for walking, and their walking is restricted outdoors and in the community; Level IV, individuals are unable to move on their own and rely on others for transportation, or use electric devices for walking outdoors and in the community; Level V, individuals require assistive technologies such as wheelchairs, and their self - movement is severely restricted.

### 2.4 Swallowing function assessment

Eating function, which directly impacts nutritional intake and rehabilitation potential in CP, was assessed using the Eating and Drinking Ability Classification System (EDACS), developed by Sellers et al. in 2014 (8) This system categorizes swallowing and eating ability based on safety and efficiency with solid and liquid foods: Level I: Eats safely and efficiently; Level II: Eats safely but with reduced efficiency; Level III: Reduced safety, possible limitations in diet; Level IV: Markedly reduced safety; Level V: Unable to eat safely; enteral feeding recommended. EDACS has demonstrated high reliability and validity for evaluating feeding abilities in children with CP.

### 2.5 Subjective global nutritional assessment

The SGNA assessment involves using scoring form to evaluate pediatric patients. This scoring form primarily consists of two parts: an inquiry section and a physical examination section. The inquiry section is composed of seven simple nutrition - related questions, including the current height relative to age, the current weight relative to height, non - targeted weight changes, adequacy of dietary intake, gastrointestinal symptoms, nutrition - related functional activity ability, and the metabolic stress of the disease. The SGNA score for each question includes three grades: normal, moderate, and severe. When scoring, factors such as the severity, the duration of the change, and recent improvements need to be considered. The physical examination section includes three items: subcutaneous fat loss (in the cheeks, arms, chest, and buttocks), muscle wasting (in the temporal bones, collarbones, shoulders, scapulae, thighs, knees, and calves), and nutrition - related edema. The score for each item is also divided into three grades: normal, moderate, and severe (9).

### 2.6 Classification of cerebral palsy

According to the characteristics of abnormal movements, cerebral palsy can be classified into five types: ① Spastic type, which is mainly characterized by damage to the pyramidal system; ② Dyskinetic type, which is primarily associated with damage to the extrapyramidal system, resulting in an increase in involuntary movements such as athetoid movements, choreiform movements, dystonia, and tremors; ③ Ataxia type, which is mainly caused by damage to the cerebellum; ④ Hypotonic type, which often serves as a transitional form to other types; ⑤ Mixed type, in which several of the above types co - exist. The clinical symptoms of each type vary in severity or are roughly the same, and most cases are a combination of the spastic and dyskinetic types. Spastic cerebral palsy is the most common type, accounting for approximately 70% of all cases. Based on the affected body parts, it can be further classified into the following five categories: ① Hemiplegia: One side of the body is affected. ② Diplegia: All four limbs are affected, with the upper limbs being less severely affected and the lower limbs being the primary site of impairment. ③ Quadriplegia: All four limbs are affected, with a similar degree of impairment in both the upper and lower limbs. ④ Monoplegia: A single limb is affected. ⑤ Triplegia: Three limbs are Affected (10).

### 2.7 Questionnaire

Parents were informed of the study's purpose and data privacy measures, and written consent was obtained. A self-designed questionnaire, developed based on region-specific and work-related common issues, was administered. A total of 58 questionnaires were distributed; responses with three or more unanswered items were deemed invalid. Ultimately, 57 valid questionnaires were collected, yielding an effective recovery rate of 98%. The survey covered four domains: (1) demographic characteristics of the children and their parents; (2) parental knowledge of cerebral palsy; (3) compliance with treatment and nutritional management; and (4) awareness of psychological well-being and pain. The questionnaire was designed for clarity, practicality, and ease of completion, making it suitable for formal investigation.

### 2.8 Malnutrition assessment

Nutritional status was assessed according to the criteria of the European Society of Pediatric Gastroenterology, Hepatology and Nutrition. Indicators of malnutrition included the following (11): weight-for-age *Z*-score (WAZ) < −2, triceps skinfold thickness below the 10th percentile for age and sex, mid–upper arm circumference below the 10th percentile, or slow weight gain. Based on the World Health Organization growth standards for children aged 0–19 years, malnutrition was classified as follows: Low weight: WAZ ≤ −2; Stunting: height-for-age *Z*-score (HAZ) ≤ −2; Wasting: weight-for-height *Z*-score (WHZ) ≤ −2; Developmental delay: HAZ ≤ −2.The malnutrition criteria in this study are based on the criteria of ASPEN: An individual is considered to have malnutrition if their weight-for-height *Z*-score (WHZ) or age-specific BMI *Z*-score (BAZ)is below −1, according to the American Society for Parenteral and Enteral Nutrition (ASPEN) consensus statement (12).

Testing instruments and testing methods. The total protein determination is carried out using the diazotization method, and the albumin determination is performed using the bromocresol green method. The instruments and equipment used are: Biossays C8. The 25-hydroxyvitamin D test is conducted using the magnetic particle chemiluminescence method. The equipment used is: New Industry MAGI UMI X8.

### 2.9 Quality control

All enrolled cases were reviewed for diagnostic accuracy and treatment planning by a deputy chief physician or higher. Surveyors were trained medical staff, and measuring devices—including skinfold calipers and weight scales—were calibrated monthly. Low-value consumables, such as measuring tapes, were replaced monthly, and damaged items were replaced as needed. Data authenticity and reliability were evaluated weekly, and newly enrolled case data were reviewed monthly by the department head.

### 2.10 Statistical analysis

The obtained data were entered and organized using Excel software. Statistical analysis was conducted using SPSS 29.0 software. The Kolmogorov - Smirnov and Shapiro - Wilk tests were employed for normality analysis. For non - normal samples, P25, P50, and P75 were used for description. For measurement data, the two - independent - sample *t*-test was used for comparison, and for non - normal measurement data, the rank - sum test for two - sample comparison was used for comparison. A *P*-value less than 0.05 indicated a statistically significant difference. The chi - square test was used to analyze the independence between categorical variables. The Kendall's Tau or Kendall Coefficient of Concordance was used as the statistic for the consistency between rank variables, and the weighted kappa was used for the consistency of ordered categorical variables.

## 3 Results

### 3.1 General characteristics of patients with cerebral palsy

A total of 58 children with cerebral palsy were enrolled (34 males, 24 females).GMFCS levels I–V included 18, 14, 11, 9, and 6 cases, respectively. Subtype distribution was as follows: spastic quadriplegia (*n* = 25), spastic hemiplegia (*n* = 19), spastic diplegia (*n* = 6), non-spastic dyskinesia (*n* = 4), and mixed type (*n* = 4) (Table 1).

**Table 1:** The GMFCS grades and types of cerebral palsy in children of different age groups.

Age	Gender		GMFCS					Types of cerebral palsy				
	Male	Female	Ⅰ	Ⅱ	Ⅲ	IV	Ⅴ	Spastic quadriplegia	Hemiplegic paralysis	Spastic diplegia	Non-ambulatory	mixed type
1–2	14	9	6	5	6	4	2	11	9	1	0	2
2–3	9	9	3	5	3	4	3	10	5	2	0	1
3–4	4	2	2	0	2	1	1	3	1	0	1	1
4–6	3	4	5	2	0	0	0	1	3	1	2	0
6–9	4	0	2	2	0	0	0	0	1	2	1	0

### 3.2 Weight/age *Z*-score for children with cerebral palsy, weight/height *Z*-score, height/age *Z*-score, BMI/Age *Z*-score

**Figure 1:**
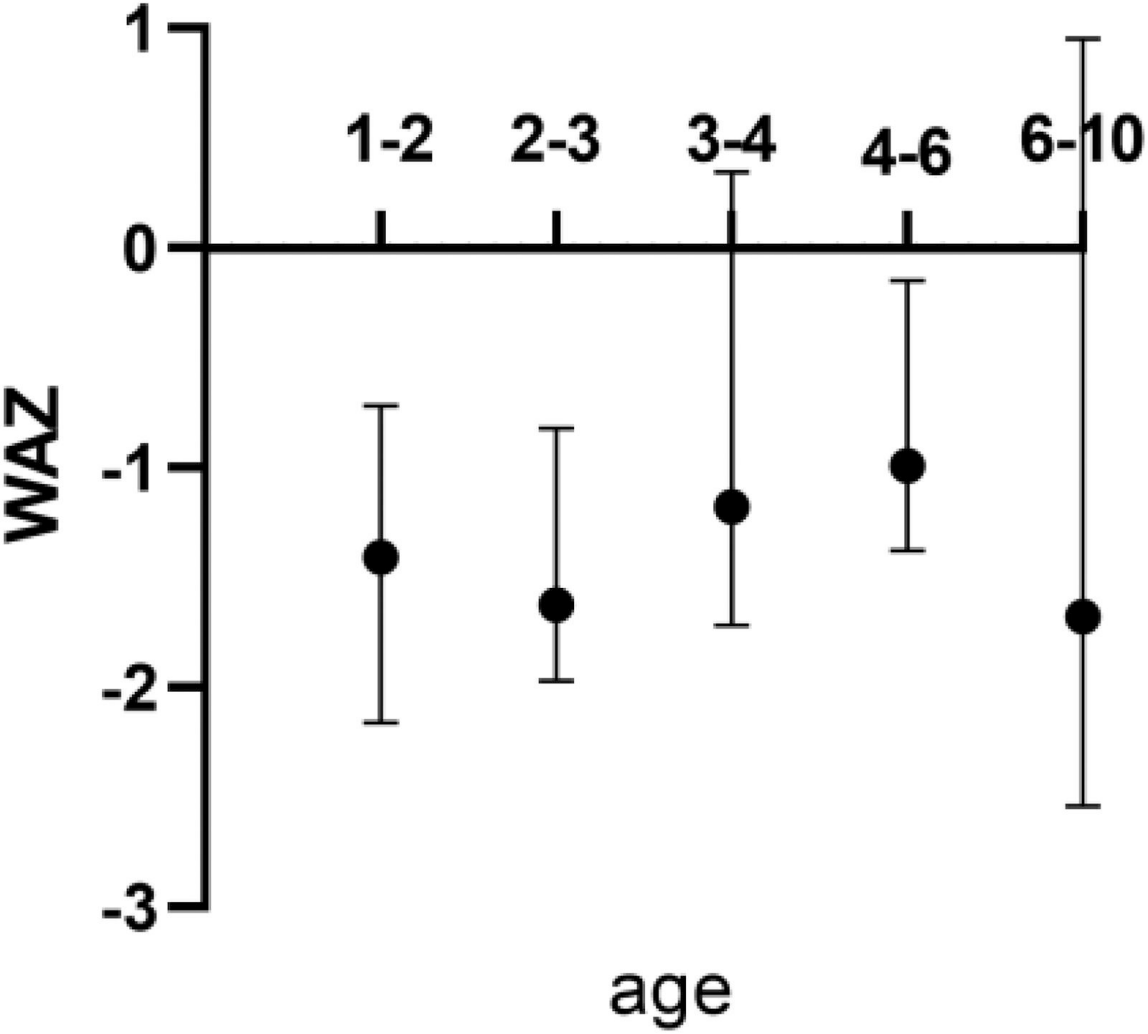
Weight/age *Z*-scores P25, P50, P75 by different age groups.

**Figure 2:**
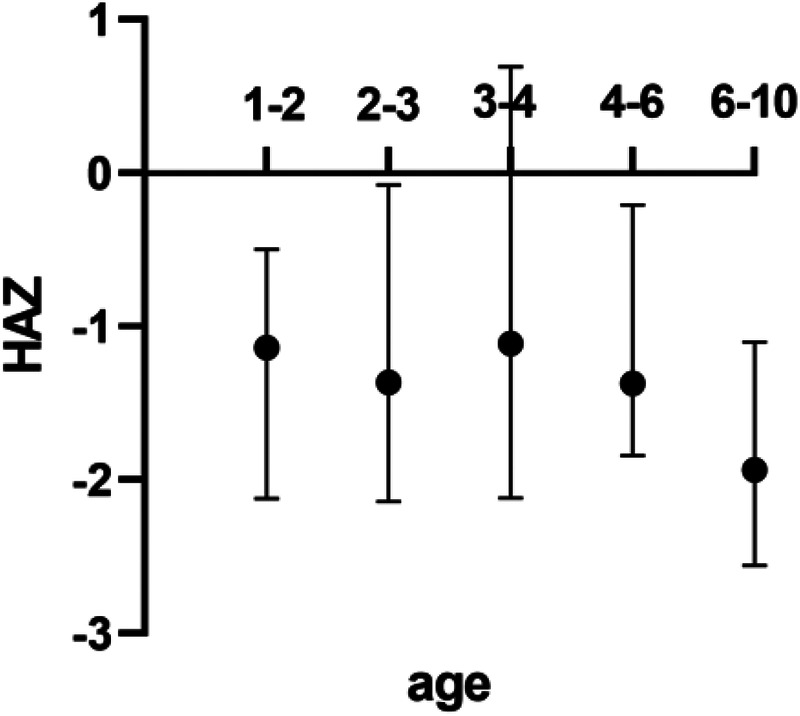
Weight/height *Z*-scores P25, P50, P75 by different age groups.

**Figure 3:**
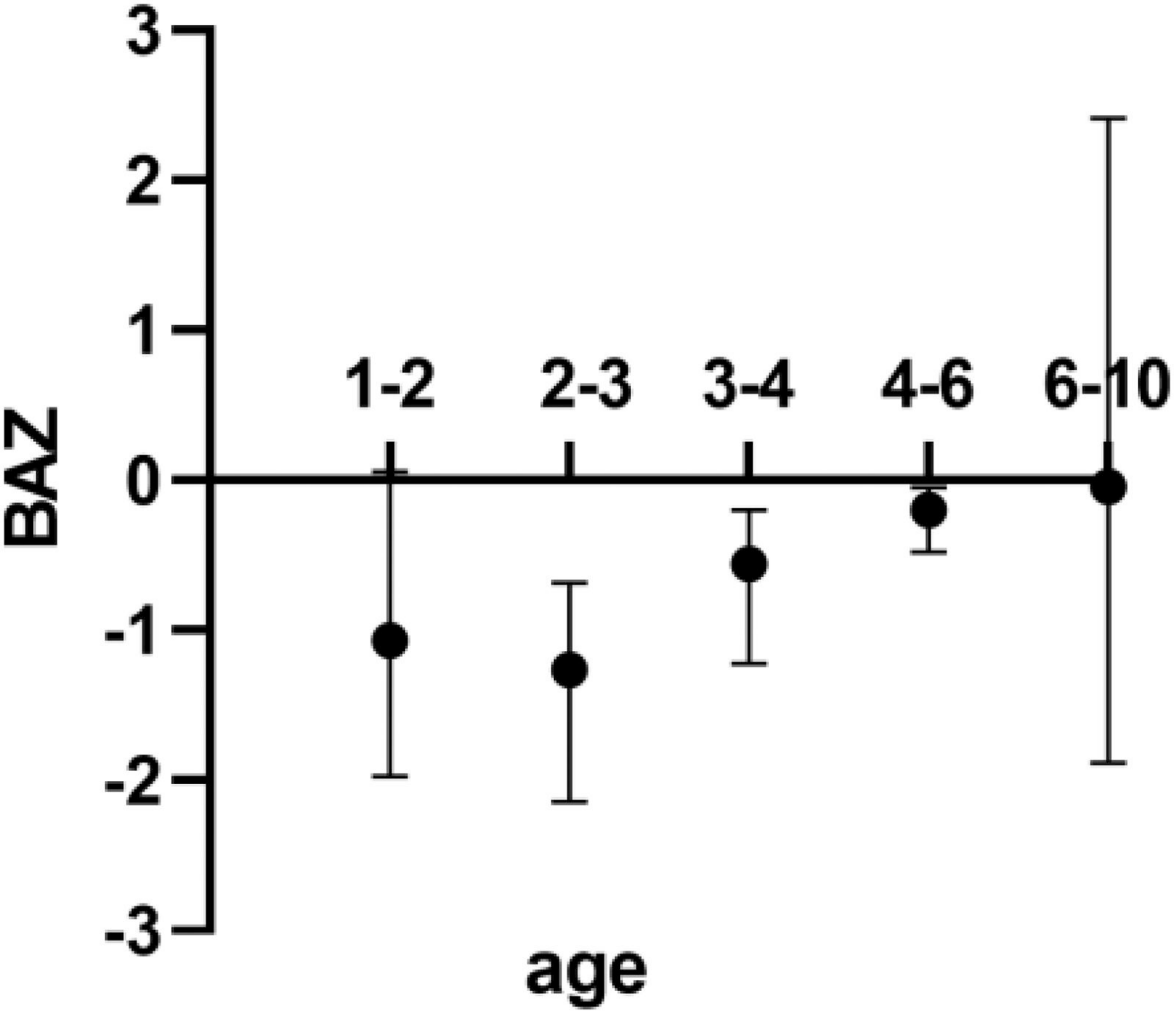
BMI/age *Z*-scores P25, P50, P75 by different age groups.

**Figure 4:**
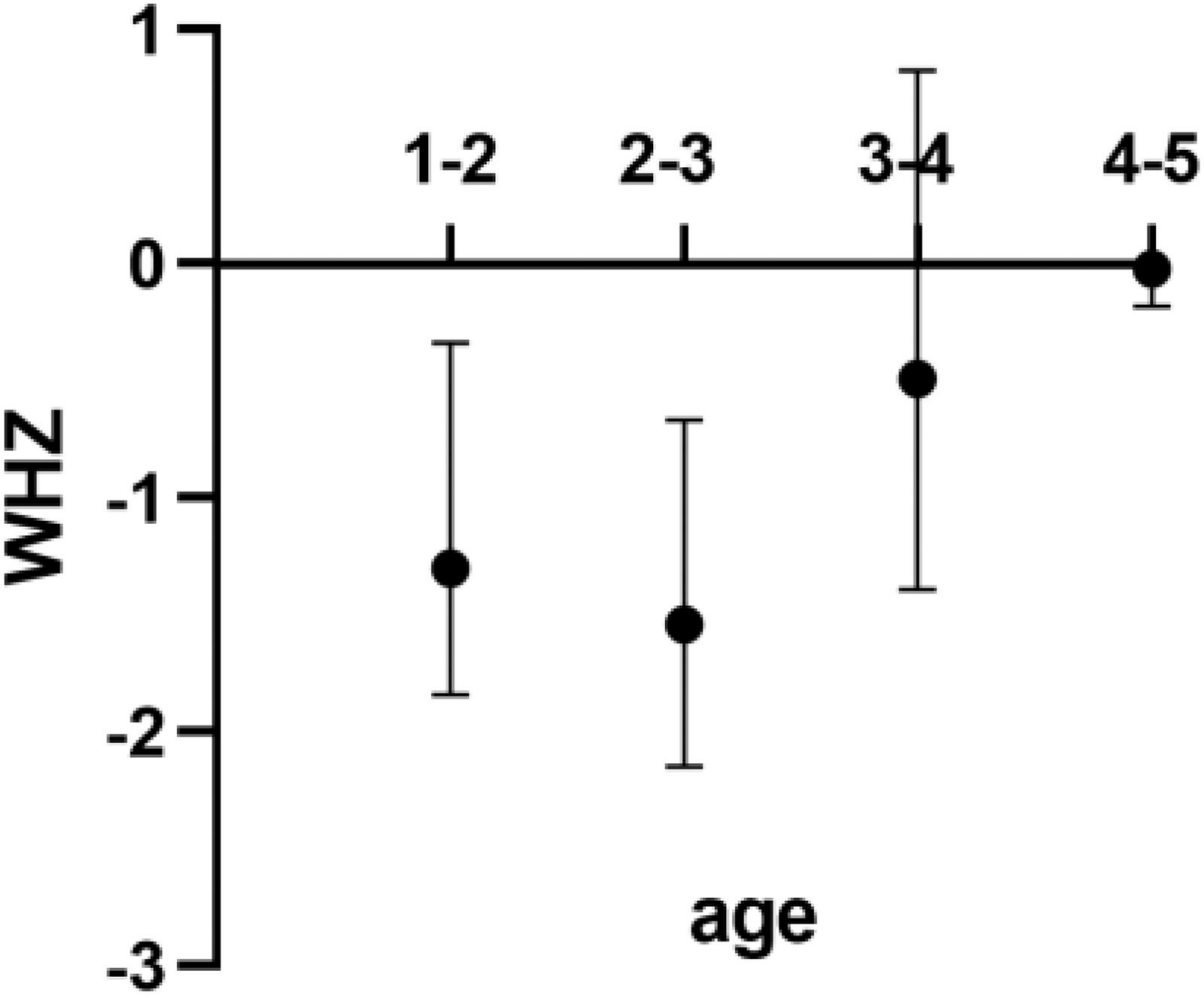
Height/weight *Z*-scores P25, P50, P75 by different age groups.

Twenty-nine children had BAZor WHZ < −1, yielding a malnutrition incidence of 50.00%. Median WAZ, WHZ, HAZ, and BAZ values were all negative and below normal reference levels, indicating varying degrees of malnutrition in all participants. Given the limited sample size, statistical conclusions require confirmation with a larger cohort (Figures 1–4).

### 3.3 Swallowing situations in children with cerebral palsy

#### 3.3.1 The prevalence of dysphagia

For analysis, EDACS, levels were dichotomized: Level I (normal eating) and levels II–V (various degrees of feeding difficulty).the prevalence of dysphagia among children with cerebral palsy was 48.27%% representing 28 confirmed cases (Table 2).

**Table 2:** The EDACS grades of children with cerebral palsy of different age groups.

EDACS	1–2 (year)	2–3 (year)	3–4 (year)	4–6 (year)	6–10 (year)
1	12	6	3	6	3
2	3	8	2	1	1
3	3	4	1	0	0
4	5	0	0	0	0
5	0	0	0	0	0
Incidence rate of dysphagia	47.82%	66.67%	50.00%	14.28%	25%
Overall rate of dysphagia	52.83%				

#### 3.3.2 A Kendall tau-b correlation analysis was conducted between EDACS level and GMFCS level

A correlation analysis was performed to examine the association between the EDACS and GMFCS levels. The results are presented in Table 3. The Kendall tau-b correlation coefficient was 0.165 (*p* = 0.14＞0.01, two-tailed), indicated EDACS level and GMFCS have no obvious correlation (Tables 3, 4).

**Table 3:** EDACS status of children with cerebral palsy at different GMFCS levels.

GMFCS	EDACS					
	Ⅰ	Ⅱ	Ⅲ	IV	Ⅴ	Total
Ⅰ	12	6	0	0	0	18
Ⅱ	4	4	3	3	0	14
Ⅲ	9	1	1	0	0	11
IV	1	4	3	1	0	9
Ⅴ	4	0	1	1	0	6
Total	30	15	8	5	0	

**Table 4:** Kendall tau-b correlation analysis between EDACS level and GMFCS level.

	correlation index	Sig (two side)	N
GMFCS	1		58
EDACS	0.165	0.141	58

### 3.4 Concordance of nutritional classification between the SGNA and ASPEN *Z*-score assessments

Nutritional classification according to the 2014 ASPEN Consensus Statement was applied to 58 children with cerebral palsy, and concordance with SGNA was assessed statistically. The ASPEN *Z*-score–based grading system defines four categories: well-nourished (Z ≥ −1), mild malnutrition (−2 < Z ≤ −1), moderate malnutrition (−3 < Z ≤ −2), and severe malnutrition (Z ≤ −3) (Table 5). To align with the three-tiered SGNA classification (well-nourished, malnourished, severely malnourished), the mild and moderate malnutrition categories were collapsed into a single “malnourished” level. Concordance between SGNA and ASPEN *Z*-score–based nutritional classification was evaluated using weighted kappa statistics. As shown in Table 6, the weighted kappa coefficient was *κ* = 0.741 (95% CI: 0.623–0.859), indicating substantial agreement beyond chance, with statistical significance at *p* < 0.001 (Table 6).

**Table 5:** SGNA and ASPEN Z malnutrition grading.

statistical value	SGNA	ASPEN Z
Normal	32 (55.17%)	29 (50%)
Moderate	22 (37.93%)	27 (46.55%)
Severe	4 (6.70%)	2 (3.44%)
The incidence of malnutrition	26 (44.82%)	29 (50%)

**Table 6:** Cohen weighted kappa of SGNA and ASPEN Z.

Cohen weighted kappa						
Grade	Weighted Kappa^a^	Asymptotic			95% asymptotic confidence interval	
		Standard error^b^	*z* ^c^	Significance	Lower limit	Upper limit
SGNA-ASPEN *Z*	0.741	0.079	6.610	<0.001	0.586	0.895

a The estimation of weighted Kappa will employ linear weights;

b The value does not depend on the null hypothesis or the alternative hypothesis;

c Under the assumption of “weighted Kappa equals zero”, estimate the asymptotic standard error.

Among the 58 children with cerebral palsy, SGNA classification indicated normal nutrition in 32 cases (55.17%) and malnutrition in 26 cases (44.82%). By contrast, ASPEN *Z*-score–based classification identified normal nutrition in 29 cases (50.00%) and malnutrition in 29 cases (50.00%). The high prevalence of malnutrition—observed in nearly half of the cohort by both assessment tools—underscores the critical need for systematic nutritional screening and targeted intervention in this population.

### 3.5 Comparison of serum total protein and albumin in children with cerebral palsy

Among the 58 children, 48 underwent serum albumin and total protein testing. A control group of 42 healthy children (matched for age and sex, *P* > 0.05) from Kunming Children's Hospital (January 2023–December 2024) was included. Serum albumin and total protein levels in the cerebral palsy group were significantly lower than controls (*P* < 0.05) (Tables 7–11). Normality testing was performed for serum albumin and total protein concentrations in both groups. Data from the healthy control group satisfied the assumption of normality, whereas values in the cerebral palsy group significantly deviated from normal distribution (Shapiro–Wilk test, *p* < 0.05). Consequently, between-group comparisons were conducted using the Mann–Whitney *U*-test. Results revealed significantly lower median serum albumin (*p* = 0.003) and total protein (*p* = 0.012) levels in children with cerebral palsy compared with healthy controls.

**Table 7:** The P25, P50, and P75 values of albumin and total protein in the normal control group and the cerebral palsy group.

Group		*N*	P25	P50	P75	Mean value	95% confidence interval (Bootstrap)	
							Lower limit	Upper limit
Albumin	Normal control group	42	42.25	43.70	45.92	43.87	43.14	44.68
	Cerebral palsy group	48	39.73	42.30	43.70	40.88	39.57	42.00
Total protein	Normal control group	42	64.53	67.40	70.85	67.87	66.51	69.27
	Cerebral palsy group	48	60.82	62.50	66.60	61.72	60.27	63.07

**Table 8:** Rank of albumin in the normal control group and the cerebral palsy group.

Group	N	Mean of squares	Sum of ranks
Normal control group	42	54.73	2,298.50
Cerebral palsy grouptotal	48	37.43	1,796.50
Total	90		

**Table 9:** Test statistic of albumin between normal control group and the cerebral palsy group.

Mann–Whitney U	Wilcoxon W	Z	Asymptotic significance (two-sided)
620.500	1796.500	−3.135	0.002

**Table 10:** Rank of total protein in the normal control group and the cerebral palsy group.

Group	N	Mean of squares	Sum of ranks
Normal control group	42	58.55	2,459.00
Cerebral palsy grouptotal	48	34.08	1,636.00
Total	90		

**Table 11:** Test statistic of total protein between normal control group and the cerebral palsy group.

Mann–Whitney U	Wilcoxon W	Z	Asymptotic significance (two-sided)
460.000	1636.000	−4.433	0.000

### 3.6 Comparison of blood 25-hydroxy-vitamin D levels in children with cerebral palsy

Serum 25-hydroxyvitamin D concentrations (nmol/L) were measured in 42 of the 58 children with cerebral palsy. For comparison, 45 age- and sex-matched healthy controls—recruited from routine physical examinations at Kunming Children's Hospital between January 2023 and December 2024—underwent identical assay procedures. Normality testing (Shapiro–Wilk test) confirmed approximate normal distribution for both groups. No statistically significant differences in age or sex distribution were observed between groups (all *p* > 0.05). Independent-samples *t*-tests revealed no statistically significant difference in mean serum 25-hydroxyvitamin D concentrations between children with cerebral palsy (*n* = 42) and healthy controls (*n* = 45) (mean difference = −3.2 nmol/L; 95% CI: −8.7–2.3; *t* = −1.16, *p* = 0.249). Of the 57 participants in the intervention group who were prescribed daily vitamin D supplementation (≥1 capsule/day), 39 (68.42%) adhered to the regimen. In the normal control group, 45 children were invited for follow-up assessment; among them, 28 (62.22%) reported consistent daily vitamin D supplementation (≥1 capsule/day). Chi-square testing showed no significant association between diagnostic group (cerebral palsy vs. control) and regular vitamin D supplementation status (χ^2^ = 0.03, df = 1, *p* = 0.862). Sunlight exposure duration was not systematically recorded for individual participants in this study, this represents a key limitation and an important target for refinement in future studies (Tables 12, 13).

**Table 12:** 25-hydroxyvitamin D of normal control group and the cerebral palsy group.

Group	*N*	Mean value	Standard deviation	Mean value	95% confidence interval (Bootstrap)	
					Lower limit	Upper limit
Normal control group	45	111.50	30.33	104.06	95.53	112.11
Cerebral palsy group total	42	104.06	28.53	113.32	104.49	122.54

**Table 13:** *t*-test of 25-hydroxyvitamin D between normal control group and the cerebral palsy group.

t	df	Sig (bilateral)	Mean difference
1.178	85	0.242	7.45

### 3.7 Compliance of family members of children with cerebral palsy

A total of 58 copies were issued, Sources of cerebral palsy–related information included medical staff (70.17%), online sources (12.28%), and other channels (17.54%). Over half of parents (52.63%) believed that cerebral palsy is associated with malnutrition, while 47.36% did not. thirty-five percent believed their child was malnourished; 26.31% had sought care from a nutrition department. Regular supplementation practices included vitamin D drops (68.42%) and calcium (52.63%). High-calorie formula milk powder was used by 26.32% of families, while 73.68% had never used it. Only 12.28% of parents reported regular nutritional assessments for their children (Table 14).

**Table 14:** Questionnaire from family members of children with cerebral palsy.

Questionnaire item	Option	Percentage (noun)
		(%)
Channels for acquiring knowledge about cerebral palsy	Medical staff education and	40 (70.17)
	Network access route	7 (12.28)
	Else	10 (17.54)
Can cerebral palsy be accompanied by malnutrition?	Yes	30 (52.63)
	No	27 (47.36)
Do you think the child is suffering from malnutrition?	Yes	20 (35.09)
	No	37 (64.91)
Have you ever visited the nutrition department?	Yes	15 (26.31)
	No	42 (73.68)
Should vitamin D drops be taken regularly?	Yes	39 (68.42)
	No	18 (31.58)
Is it necessary to regularly take calcium supplements?	Yes	30 (52.63)
	No	27 (47.37)
Have you ever added high- calorie formula milk?	Yes	15 (26.32)
	^No^	42 (73.68)
Is there a regular nutritional assessment conducted (once every 1–3 months)?	Yes	7 (12.28)
	No	50 (87.22)

## 4 Discussion

### 4.1 Nutritional status of children with cerebral palsy

Internationally, the nutritional challenges faced by children with CP remain a major concern. Reported malnutrition rates vary widely: approximately 7.90% in the United States (13), 57.20% (4) in Turkey, 81.40% (14) in Malaysia, and 85.90% (15) in Nigeria. Strand et al. assessed 104 children with CP aged 0–5 years and found significantly lower nutritional levels compared to baseline values for healthy peers. Height and weight indices declined markedly before age two, then stabilized between ages two and five. Children with milder motor impairment had significantly higher growth indices than those with severe CP (16). Similarly, Penagini et al. (17) reported that 46.00%–90.00% of children with CP experienced malnutrition. In China, the incidence of malnutrition in CP remains high, yet systematic and standardized nutritional intervention studies are scarce. Li Meirui reported a malnutrition rate of 48.97%, with 26.53% of cases classified as underweight (18)—significantly higher than rates observed in healthy children. Wang Jun's study found that moderate to severe underweight affected 31.5% of children with CP, growth retardation occurred in 39.70%, and wasting in 21.90%. The Gross Motor Function Classification System (GMFCS) level and swallowing disorders were identified as risk factors for malnutrition (19). In this study, the prevalence of nutritional deficiency among children with cerebral palsy defined per the 2014 ASPEN *Z*-score–based criteria was 50.00%. Variability in nutritional status across individuals may reflect regional disparities in healthcare access, nutritional support infrastructure, and socioeconomic determinants of health, contributing to heterogeneity in reported prevalence rates across global studies. Participants were stratified into five age groups (1–2, 2–3, 3–4, 4–6, and 6–9 years), and weight-for-age, weight-for-height, height-for-age, and BMI-for-age *Z*-scores were calculated. Due to non-normal data distribution, quartiles were used to describe central tendency and dispersion. All median *Z*-scores were negative and below normal, indicating that all 58 participants experienced some degree of malnutrition. Swallowing function was evaluated using the Eating and Drinking Ability Classification System (EDACS). The prevalence of dysphagia was 48.27%%, consistent with findings by Chen Jing (20), who assessed 81 children with CP and reported that over half (44 cases) had EDACS grade II or higher. Dysphagia in CP is likely due to central nervous system damage, which impairs early motor function development, particularly in the oropharyngeal region, thereby delaying the acquisition of feeding skills during infancy. Chen Jing (20) reported a weak positive correlation between GMFCS level and EDACS score. In contrast, the present study based on a smaller sample (*n* = 58) detected no statistically significant association (*r* = 0.12, *p* = 0.364). Given the limited statistical power arising from the sample size, these findings do not refute Chen Jing's observation but preclude definitive conclusions regarding the strength or direction of the relationship. A larger, adequately powered study is warranted to clarify this association.

With regard to nutritional assessment, the present study demonstrates substantial agreement between ASPEN *Z*-score–based classification and SGNA classification (weighted *κ* = 0.741), consistent with findings reported by Chen Jing (20). Nutritional evaluation in children with cerebral palsy is inherently more complex than in neurotypical peers: underlying neurological impairment may alter physiological responses including growth patterns, inflammatory markers, and functional capacity, thereby confounding conventional anthropometric and biochemical indicators and necessitating integrated, condition-specific assessment frameworks. Anthropometric *Z*-score calculation while widely used is operationally demanding and prone to measurement error in children with cerebral palsy, particularly due to postural deformities, contractures, and difficulties in obtaining reliable height/length measurements. Moreover, the marked clinical heterogeneity across GMFCS levels, motor subtypes, and comorbidities renders the selection of an appropriate reference growth standard inherently challenging; no universally validated “cerebral palsy–specific growth curve” currently exists. In contrast, the Subjective Global Nutritional Assessment (SGNA) offers a pragmatic, clinically integrated alternative. Empirical evidence including the present study and prior work by Chen Jing (20) demonstrates its validity for nutritional assessment in this population and strong agreement with *Z*-score–based classification (weighted *κ* = 0.741). Consequently, SGNA is recommended as a core component of routine nutritional screening for children with cerebral palsy.

### 4.2 Vitamin and protein levels

Biochemical analysis revealed that serum albumin and total protein levels in the CP group were significantly lower than in healthy controls (*P* < 0.05). This may be attributed to feeding difficulties such as poor sucking, coughing during feeding (21), and swallowing dysfunction, combined with reduced voluntary physical activity, all of which limit dietary intake. Vitamin D (VD) plays a critical role in multiple organ functions and significantly contributes tocardiac metabolism and musculoskeletal health (22). The primary circulating form, 25-hydroxyvitamin D, serves as a reliable biomarker for assessing VD status due to its relatively long half-life, ability to reflect both dietary intake and endogenous synthesis, and stability *in vivo* (23) In childhood, adequate 25-hydroxyvitamin D levels are essential for bone growth, mineralization, and overall musculoskeletal development. Severe deficiency can result in rickets, metabolic bone disease, and hypocalcemia during growth. Several studies have reported significantly lower serum 25-hydroxyvitamin D levels in children with cerebral palsy (CP) compared to typically developing peers, often attributed to reduced sunlight exposure and limited physical activity. However, in the present study, no statistically significant difference in serum 25-hydroxyvitamin D levels was observed between children with CP and controls. Possible explanations include: (1) consistent vitamin D supplementation practices across groups—68.4% of children with cerebral palsy and 62.2% of healthy controls reported regular daily intake (≥400 IU/day), with no statistically significant difference between groups (χ^2^ = 0.03, df = 1, *p* = 0.862); and (2) unmeasured environmental influences, particularly variability in habitual sunlight exposure and geographic UV index differences, which were not systematically assessed in this study and warrant prospective investigation in future research.

### 4.3 Questionnaire

In this study, the prevalence of malnutrition among children with CP was 50%. Despite this, questionnaire results revealed gaps in caregiver awareness 47.36% of families believed CP was not associated with malnutrition, 64.91% did not consider their child malnourished, 73.68% had never sought nutrition department consultations, and 87.72% had not obtained regular nutritional assessments. These findings indicate low caregiver awareness and poor adherence to nutritional management recommendations.

Given the high incidence of malnutrition, improving caregiver compliance is essential.

Recommended strategies include strengthening parental health education, implementing widespread nutrition education programs for caregivers, improving feeding knowledge and skills, and ensuring regular professional nutritional assessments. Simplifying nutritional assessment procedures for healthcare providers may further enhance the nutritional outcomes of children with CP.

## 5 Conclusions

In this study, we found that children with cerebral palsy have a relatively high incidence of malnutrition. However, the compliance of family members regarding the nutritional management of children with cerebral palsy is relatively low. Strengthening health education for parents, actively and extensively implementing nutritional education services and training programs for caregivers of children with cerebral palsy, improving caregivers' feeding knowledge and skills, instructing them to undergo regular professional nutritional assessments, and simplifying the nutritional assessment process for medical workers are crucial for improving the nutritional status of children with cerebral palsy.

## 6 Limitations

This study was conducted at a single-center, and the sample size was relatively small. The result cannot be directly used for making inferences about the overall situation. The source of patients with cerebral palsy is limited, which restricts the collection of data., Future studies should consider expanding the sample size, stratified random sampling, conducting multi-center collaborations to enhance the generalizability of the findings. Additionally, efforts should be made to include a more diverse cohort of cerebral palsy patients, covering different age groups, severity levels, and etiological subtypes, to capture a broader spectrum of the condition. Moreover, the current study lacked long-term follow-up data to assess the sustained effects of the intervention; future research should incorporate extended follow-up periods to provide more comprehensive insights into the outcomes. Additionally, the study did not account for potential confounding factors such as comorbidities or socioeconomic status, which might influence the results; future investigations should control for these variables to strengthen the causal inferences.

## Data Availability

The datasets presented in this study can be found in online repositories. The names of the repository/repositories and accession number(s) can be found in the article/Supplementary Material.

## References

[1] Dan B Rosenbaum P Carr L Gough M Coughlan J Nweke N. Proposed updated description of cerebral palsy. Dev Med Child Neurol. (2025) 67(6):700–9. 10.1111/dmcn.16274 40213981

[2] McGuire DO Tian LH Yeargin-Allsopp M Dowling NF Christensen DL. Prevalence of cerebral palsy, intellectual disability, hearing loss, and blindness, national health interview survey, 2009–2016. Disabil Health J. (2019) 12(3):443–51. 10.1016/j.dhjo.2019.01.005 30713095 PMC7605150

[3] Feng Y Pang W Li X Yang S Liu S Lu S. Meta-analysis of the prevalence of cerebral palsy in children aged 0–6 years in China [J]. Chin Gen Pract. (2021) 24(5):5. 10.12114/j.issn.1007-9572.2021.00.072

[4] Aydın K Kartal A Keleş Alp E. High rates of malnutrition and epilepsy: two common comorbidities in children with cerebral palsy[J]. Turk J Med Sci. (2019) 49(1):33–7. 10.3906/sag-1803-79 30761843 PMC7350856

[5] Leonard M Dain E Pelc K Dan B De Laet C. Nutritional status of neurologically impaired children: impact on comorbidity[J]. Arch Pediatr. (2020) 27(2):95–103. 10.1016/j.arcped.2019.11.003 31791829

[6] Caselli TB Lomazi EA Montenegro M Bellomo-Brandao MA. Assessment of nutritional status of children and adolescents with spastic quadriplegic cerebral palsy. Arq Gastroenterol. (2017) 54(3):201–5. 10.1590/S0004-2803.201700000-32 28723982

[7] McDowell B. The gross motor function classification system—expanded and revised[J]. Dev Med Child Neurol. (2008) 50(10):725–30. 10.1111/j.1469-8749.2008.03104.x 18834382

[8] Tschirren L Bauer S Hanser C Marsico P Sellers D van Hedel HJA. The eating and drinking ability classification system: concurrent validity and reliability in children with cerebral palsy. Dev Med Child Neuro. (2018), 60(5):611–7. 10.1111/dmcn.13751 29656386

[9] Peng T Zhao Y Fu C Yanjie W. A study of validity and reliability for subjective global nutritional assessment in outpatient children with cerebral palsy[J]. Nutr Neurosci. (2021) 24(7):1–7. 10.1080/1028415X.2021.1990463 34663203

[10] Kaishou XU. Pediatric Physical Therapy [M]. Sun Yat-sen University Press. Guangzhou: Guangzhou Sun Yat-sen University Press. (2016), 312.

[11] Romano C van Wynckel M Hulst J Broekaert I Bronsky J Dall'Oglio L. European Society for paediatric gastroenterology, hepatology and nutrition guidelines for the evaluation and treatment of gastrointestinal and nutritional complications in children with neurological impairment. J Pediatr Gastroenterol Nutr. (2017) 65(2):242–64. 10.1097/MPG.0000000000001646 28737572

[12] Becker P Carney LN Corkins MR Goday PS Krebs NF Lenders LB. Consensus statement of the academy of nutrition and dietetics/American society for parenteral and enteral nutrition: indicators recommended for the identification and documentation of pediatric malnutrition (undernutrition). Nutr Clin Pract. (2015) 30(1):147–61. doi: 10.1177/0884533614557642 25422273 10.1177/0884533614557642

[13] Reyes FI Salemi JL Dongarwar D Magazine CB Salihu HM. Prevalence, trends, and correlates of malnutrition among hospitalized children with cerebral palsy. Dev Med Child Neurol. (2019) 61(12):1432–8. 10.1111/dmcn.14329 31378936

[14] Ahmad R Rahman NA Hasan R Yaacob NS Ali SH. Oral health and nutritional status of children with cerebral palsy in northeastern peninsular Malaysia. Spec Care Dent. (2019) 40(1):62–70. 10.1111/scd.12436 31774579

[15] Adamu AS Sabo UA Gwarzo GD Belonwu RO. Nutritional status in cerebral palsy:a cross-sectional comparative survey of children in kano, Nigeria. Niger Postgrad Med J. (2018) 25(3):156–60. 10.1111/scd.12436 30264766

[16] Strand KM Dahlseng MO Lydersen S Rø TB Finbråten A Jahnsen RB. Growth during infancy and early childhood in children with cerebral palsy: a population-based study. Dev Med Child Neuro. (2016) 58(10):924–30. 10.1111/dmcn.13098 26992128

[17] Penagini F Mameli C Fabiano V Brunetti D Dilillo D Zuccotti GV. Dietary intakes and nutritional issues in neurologically impaired children. Nutrients. (2015) 7(12):9400–15. 10.3390/nu7115469 26580646 PMC4663597

[18] Meirui L Jianmin W Honghong Z Jie M Qiaoyu C Chenlu X. Nutrition status and intervention effects of children with cerebral palsy. Chin Rehabil Theory Pract. (2014) 20(12):1150–2. 10.3390/nu7115469

[19] Jun W Mengyue L Dengna Z Jianghua D Yongbin L Tong P. Cross-sectional study on nutritional status and quality of life of children with cerebral palsy. Matern Child Health J. (2018) 9:31–5. 10.3969/j.issn.1006-9771.2014.12.013

[20] Jing C Guanglei T. Analysis of feeding function grading system and malnutrition in children with cerebral palsy. Chin J Child Health Care. (2022) 30(5):1150–2.

[21] Xiulan B. Early diagnosis and intervention treatment of cerebral palsy. J Pract Pediatr Clin. (2003) 18(3):160–2. 10.3969/j.issn.1003-515X.2003.03.002

[22] Doğan Y Kara M Culha MA Özçakar L Kaymak B. The relationship between vitamin D deficiency, body composition, and physical/cognitive functions. Arch Osteoporos. (2022) 17(1):66. 10.1007/s11657-022-01109-6 35420317 PMC9008297

[23] Toopchizadeh V Barzegar M Masoumi S Jahanjoo F. Prevalence of vitamin D deficiency and associated risk factors in cerebral palsy a study in north-west of Iran. Iran J Child Neurol. (2018) 12:25–32. 10.1007/s11657-022-01109-6 29696043 PMC5904735

[24] Xiaojie L Jiulai T Bingxiang M. China’s guide to rehabilitation of cerebral palsy chapter: introduction. Chin J Pract Pediatr Clin. (2022) 37(12):887–92. 10.3760/cma.j.cn101070-20220505-00500

[25] Li XJ Tang JL Ma BX. Chinese Guidelines for rehabilitation of cerebral palsy. Chin J Appl Clin Pediatr. (2022) 37(12):887–92. 10.3760/cma.j.cn101070-20220505-00500

